# Neurocognitive profile of adults with the Norrbottnian type of Gaucher disease

**DOI:** 10.1002/jmd2.12262

**Published:** 2021-11-21

**Authors:** Panagiota Tsitsi, Ioanna Markaki, Josefine Waldthaler, Maciej Machaczka, Per Svenningsson

**Affiliations:** ^1^ Department of Clinical Neuroscience Karolinska Institutet Stockholm Sweden; ^2^ Center for Neurology Academic Specialist Center Stockholm Sweden; ^3^ Department of Neurology University Hospital Marburg Marburg Germany; ^4^ Department of Human Pathophysiology Institute of Medical Sciences, University of Rzeszów Rzeszów Poland; ^5^ Department of Clinical Science and Education, Division of Internal Medicine, Södersjukhuset Karolinska Institutet Stockholm Sweden; ^6^ Department of Medicine Sunderby Hospital Luleå Sweden; ^7^ Department of Neurology Karolinska University Hospital Stockholm Sweden

**Keywords:** attention, cognition, glucocerebrosidase, memory, neuronopathic Gaucher disease

## Abstract

**Introduction:**

Gaucher disease (GD) is a monogenic, lysosomal storage disorder, classified according to the presence of acute (type 2), chronic (type 3), or no (type 1) neurological manifestations. The Norrbottnian subtype of neuronopathic GD type 3 (GD3) is relatively frequent in the northern part of Sweden. It exhibits a wide range of neurological symptoms but is characterized by extended life expectancy compared to GD3 in other countries. The aim of our study was to describe the cognitive profile of adult patients with Norrbottnian GD3.

**Materials and Methods:**

Ten patients with GD3 (five males and five females) underwent neurocognitive testing with the Repeatable Battery for Assessment of Neuropsychological Status (RBANS). RBANS consists of different short tests that assess Immediate Memory, Visuospatial and Constructional function, Language, Attention, and Delayed Memory. General neurological symptoms of the patients were assessed with the modified severity scoring tool.

**Results:**

Patients (median age 41.5 range 24–57) performed lower than average in all cognitive domains. The overall index score was low (median 58.5, Interquartile range [IQR] 25.5), with the most profound deficit in attention (median 57, IQR 32.5) and immediate memory (median 76.5, IQR 13). Higher scores were found in language (median 83, IQR 21.5), delayed memory (median 81, IQR 41), and visuospatial/constructional function (median 86, IQR 32.35).

**Conclusion:**

Norrbottnian GD3 patients showed a unique neurocognitive profile with low overall performance, mostly derived from low scores in attention and memory domains whereas language and visuospatial/constructional ability were relatively spared.

**Synopsis**

Norrbottnian Gaucher type 3 patients show deficits in attention and immediate memory but have a relatively spared language and visuospatial/constructive ability.

## 1 INTRODUCTION

Gaucher disease (GD) is caused by decreased glucocerebrosidase (GCase) activity that leads to the accumulation of glucosylceramide in monocytes and macrophages.^1^, ^2^ Three clinical subtypes are described: a non‐neuronopathic form (type 1), an acute (type 2), and a chronic neuronopathic form (type 3).^2^ All subtypes are characterized by hematological, visceral, and bone manifestations, whereas the clinical phenotypes of GD2 and 3 also present with gaze palsy, and may include symptoms from the central nervous system (CNS).^2^, ^3^, ^4^, ^5^ Other neurological features like ataxia, myoclonus, epilepsy, and dystonia‐like hyperkinetic symptoms may vary among patients.^6^ There is a relatively high prevalence (1:17.500) of a GD3 subtype in northern Sweden, the Norrbottnian type of GD3.^7^ Most of these patients are homozygotes for the missense mutation L444P (c.1448T > C) in the glucosylceramidase beta gene (*GBA*),^8^ they receive enzyme replacement therapy (ERT) and have an extended life expectancy, not only compared to other GD3 patients when left untreated,^9^ but also probably due to the availability and good response to treatment.^10^

The pathophysiology of CNS involvement in neuronopathic GD is poorly understood. Glucosylceramide accumulates rarely in neurons^2^ but the perivascular accumulation of Gaucher cells^11^ and the role of glucosylsphingosines has been studied.^2^, ^12^ Interestingly, glucosylsphingosines in the brain are involved in neuronal development and axonal growth.^13^ The role of gangliosides in neuronal repair and plasticity has previously been discussed and their abnormal accumulation in the CNS has been related to neurodevelopmental diseases and neurodegeneration.^14^ Neuronal loss in the hippocampal CA2‐4 area and the cerebral cortical layers 3 and 5 have been described in a series of seven patients with neuronopathic GD.^11^ The current therapeutic options include ERT and substrate reduction therapy (SRT), none of which is efficacious against neurological symptoms, although some SRT crosses the blood–brain barrier.^1^, ^2^ Allogeneic hematopoietic stem cell transplantation (allo‐HSCT) has been implemented in a few GD2 and GD3 cases,^15^ but it is not part of the standard care.

Current knowledge on CNS involvement is mainly based on epidemiological studies. Neurological signs are often present early in utero and infancy in GD2, while in GD3 they present later in life.^1^ Previous studies on the psychomotor and intelligence quotient (IQ) scores of pediatric GD3 patients have shown that overall intellectual delay is not common early in the disease course, but increases through childhood and adolescence and may be aggravated by splenectomy.^16^ However, a more thorough, longitudinal evaluation of cognition in GD3 patients, both children and adults, showed that the variation of IQ in GD3 is not linear, and shows no clear trajectory.^17^In a series of adult GD3 patients, only mild cognitive impairment was reported in the subgroup of those with concurrent epilepsy, as assessed with the montreal cognitive assessment screening tool.^4^, ^6^, ^16^

The objective of this study was to assess the cognitive profile of Norrbottnian GD3 patients in Sweden with the Repeatable Battery for Assessment of Neuropsychological Status (RBANS).

## 2 MATERIALS AND METHODS

This was a cross‐sectional study including 10 patients with GD3 diagnosis who are followed at the Department of Medicine, Sunderby Regional Hospital of Norrbotten County in Luleå, Sweden. All participants provided oral and written informed consent in accordance with the Helsinki Declaration and the study was approved by the local ethics committee (DNR 2016/19‐31/1 and 2017/1957‐32/1, Regionala Etikprövningsnämnden). Information on age, gender, clinical history (genotype, age at diagnosis, epilepsy, surgical treatment) as well as past and current treatments were recorded. The patients underwent clinical evaluation with the modified severity scoring tool which has been developed for the assessment of neurological manifestations in neuronopathic GD patients.^18^ Additionally, the patients underwent cognitive testing with the RBANS battery that comprises five subtests assessing immediate memory (list learning, story memory), visuospatial and constructional function (figure copy, line orientation), language (picture naming, semantic fluency), attention (digit span, coding) and delayed memory (list recall, list recognition, story recall, figure recall). Each sum score generates an index score which is scaled according to age. Index values were acquired from normative data from healthy populations, where a mean value of 100 and a SD of 15 are established as the normal range.^19^ Index score values of 90–109 are classified as average, 80–89 as the lower part of the average, 70–79 as clearly below average, and ≤69 significantly below average. The scores were computed according to the manufacturer's scoring sheet and compared to the normative data for the Swedish population. RBANS has previously been used in studies on neurodegenerative^20^ and neurovascular diseases^21^, ^22^ with relatively good reliability. It is easy to administer, takes approximately 30 min to complete, and the measurements provide a scaled‐score profile adjusted for age and education.

Eye‐tracking parameters including latency, gaze, and velocity (average and peak) of horizontal and vertical saccades, and antisaccades, as well as antisaccadic error rate, were measured with EyeBrain T2 as previously described^5^ and were available in eight patients.

### 2.1 Statistical analysis

Continuous variables are reported in medians and interquartile ranges, and categorical variables in percentages and absolute numbers. The Mann–Whitney *U* test was used for between‐group comparisons of continuous variables, and the significance level was 0.05. IBM SPSS Statistics 25 software was used for statistical analysis.

## 3 RESULTS

**FIGURE 1:**
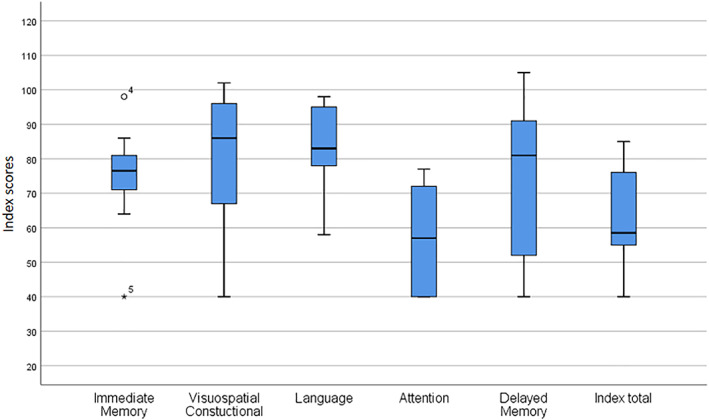
Boxplots of the RBANS index scores in five cognitive domains for the GD3 cohort. The boxplots represent the minimum, first quartile, median, third quartile and maximum of the RBANS subscores. The asterisk (*) represents an extreme outlier while the circle (o) represents a mild outlier

The GD3 cohort included five males and five females that were, all but one, homozygotes for the L444P mutation. Overall demographic and clinical characteristics of patients, as well as index scores in cognitive domains as assessed with RBANS, are summarized in Table 1. No siblings were included in the group, neither any close family relationship was reported among participants. The heterozygote (L444P/A341T) presented with milder symptoms and a better cognitive profile. In the neuropsychological assessment, patients scored at the lower part of the average in visuospatial/constructional, language, and delayed memory domains. Immediate memory score was clearly below average whereas attention, as well as the total index score, were significantly below average (Table 1 and Figure 1).

**TABLE 1:** Demographic and clinical characteristics of patients, and index scores in cognitive domains as assessed with RBANS

	Sex	Age	*GBA* genotype^a^	Age at Dx	SPC/Age	Epilepsy/ Age at Dx	Therapy/Age	mSST	Immediate Memory	Visuospatial/ Constructional	Language	Attention	Delayed Memory	Total Index Score
1	F	32	c.[1448 T > C];[1448 T > C]	2	Y/2	Y/16	allo‐HSCT/2	7.5	71	90	82	59	52	56
2	F	39	c.[1448 T > C];[1448 T > C]	7	Y/10	N	ERT/13	5	75	56	82	67	80	58
3	M	29	c.[1448 T > C];[1448 T > C]	2	Y/3	Y/17	ERT/5	11.5	86	102	95	55	91	79
4	M	52	c.[1448 T > C];[1448 T > C]	5	Y/13	Y/45	ERT/27	14	98	82	88	77	105	85
5	F	44	c.[1448 T > C];[1448 T > C]	2	Y/8	Y/23	allo‐HSCT/9	18	40	67	58	40	40	40
6	M	30	c.[1448 T > C];[1448 T > C]	3	N	Y/14	ERT/4	15	75	90	60	40	85	55
7	F	51	c.[1448 T > C];[1448 T > C]	1	Y/19	N	ERT/26	5.5	78	70	95	40	80	59
8	F	57	c.[1448 T > C];[1448 T > C]	3	Y/3	N	ERT/32	12.5	81	98	84	74	82	76
9	M	51	c.[1448 T > C];[1448 T > C]	1	Y/10	N	ERT27	13.5	64	40	98	40	44	40
10	M	24	c.[1138G > A];[1448 T > C]	1	N	N	ERT/1	1.5	78	96	78	72	91	75
Median (IQR)		41.5 (21.5)		2 (3)	9 (7)			12 (8.88)	76.5 (13)	86 (32.25)	83 (21.5)	57 (32.5)	81 (41)	58.5 (25.5)

Abbreviations: allo‐HSCT, allogeneic hematopoietic stem cell transplantation; Dx, diagnosis; ERT, enzyme replacement therapy; F, Female; M, Male; mSST, modified severity scoring tool; N, no; SPC, splenectomy; Y, yes.

^a^ *GBA* genotyping performed in 2015 (DNA extraction, PCR and sequencing of all coding exons and flanking intronic regions) with the exception of two patients who underwent allo‐HSCT. Median scores and IQR are computed.

Comparisons of cognitive performance between patients with and without splenectomy did not show statistically significant differences. With regard to treatment, all cognitive domain scores were lower in patients that had undergone allo‐HSCT compared to those treated with ERT (Table 2), especially immediate (median 55.5 vs. 78; *p* = 0.09) and delayed memory (median 46 vs. 83.5; *p* = 0.09). Differences did not reach statistical significance presumably due to lack of power. Finally, no statistically significant difference was found in the RBANS scores between patients with and without a diagnosis of epilepsy.

**TABLE 2:** Saccade's characteristics in Norrbottnian Gaucher disease type 3 patients

Latency (ms)	Median (IQR)
Step horizontal	293.75 (77.94)
Step downward	276.25 (63,13)
Step upward	266 (0)
Gap horizontal	251.5 (98.75)
Antisaccades (with gap)	261 (27.25)
*Gain*	
Step horizontal	0.86 (0.09)
Step downward	0.9 (0.09)
Step upward	0.86 (0.26)
Gap horizontal	0.88 (0.14)
Antisaccades error rate	0.375 (0.54)

*Note*: Median and Interquartile ranges (IQR) of eye‐movement parameters. Latency is computed in milliseconds. The gain describes the amplitude of the saccades relative to the amplitude of the visual target which was presented 20° lateral of a central fixation cross.

No significant correlations between the total and the subtest scores of the RBANS and the eye‐movement parameters were identified. Saccades' characteristics have been described in detail in a previous publication of our group,^5^ and are summarized in Table 2. In short, horizontal saccades were characterized by reduced gain, peak, and average velocity compared to healthy controls while during downwards vertical saccades, the average velocity was decreased. Latencies during both vertical and horizontal saccades were prolonged.

## 4 DISCUSSION

Here, we report the presence of significant cognitive deficits in adult patients with Norrbottnian GD3, more profound in the domains of attention and immediate memory, and milder in visuospatial/constructional function, language, and delayed memory, as assessed with the RBANS test. These results are partly in accordance with a previous study^23^ that has shown that nonverbal skills were typically affected in young patients with GD3. The study population examined presented with slow processing speed and poor performance in visuospatial, and perceptual organization skills, whereas language skills were spared. Similarly, in our population, we report a profound attention deficit and average language performance. Contrary to this study, we report relatively good performance in visuospatial and constructional tasks. This discrepancy may be due to differences in the age and genetic background of the investigated populations or might be attributed to different methodologies and cognitive assessment tools used. Additionally, results from the largest, longitudinal study on GD3, where both children and adults were extensively evaluated with age‐appropriate cognitive tests, has also highlighted a low performance in attention, as measured with digit span and coding.^17^ Moreover, the researchers highlight better scores in verbal tests compared to performance items, and indicate that this could be attributed to the fact that some of the performance subtests where timed, along with the disadvantage of impaired fine motor skills and horizontal gaze tracking. Despite the different study‐design compared to our cohort, a similar cognitive profile was observed. However, the only timed subtest in RBANS is coding (part of the performance IQ assessment in the aforementioned study), which is combined with digit span (part of the verbal IQ assessment) in order to provide a common index score for attention. Therefore, direct comparison between the studies' results is hard to be made.

There have been reports that splenectomy is associated with lower IQ and a worsening of neurological symptoms and cognitive profile in GD3 patients, probably due to accumulation of glycosylceramide in various tissues and the brain.^16^, ^23^ Our study could not confirm any statistically significant differences between the patients who had undergone splenectomy and those who had not, neither in the cognitive domains examined by RBANS nor in the total score. It has to be pointed out though that only two of the patients in our cohort were not splenectomized, while one of them is not homozygotic for the L444P mutation but has a unique genotype of L444P/A431T and a somehow milder phenotype. The results should, therefore, be interpreted with caution.

With regard to the effect of treatment on cognitive performance, patients who had undergone allo‐HSCT had more pronounced memory impairment than those who received ERT. Previous studies have not confirmed a beneficial effect of ERT against neurological manifestations of GD,^24^ however, based on smaller studies, a possible positive effect of ERT in cognitive outcome has been suggested.^25^, ^26^, ^27^ Also, allo‐HSCT is not common in clinical praxis and has, so far, been performed in a limited number of GD3 patients, with questionable effects on the neurological outcome.^28^ On the contrary, allo‐HSCT is indeed shown to affect cognition and lead to cognitive impairment in patients with hematologic malignancies.^29^ Given that treatment options are based on symptom severity, among other factors, it is difficult to draw conclusions on the nature of the relationship between treatment and outcome within such a small group of patients.

Half of the patients were treated with antiepileptic drugs, however, no significant differences were found in the cognitive performance of those with versus without epilepsy, as it was also shown in a previous study.^17^ This observation opposes previous case reports,^30^, ^31^ and a longitudinal cohort of 12 Norrbottnian GD3 patients, where three out of four patients with MoCA score lower than the cut‐off for cognitive impairment had epilepsy.^6^ Although evidence suggests that cognitive deficits are common features in patients with epilepsy and sometimes present early or before epilepsy onset,^32^ there are no systematic studies on the association of epileptic features in GD with cognitive performance.

We were not able to identify statistically significant correlations between the eye movements and the RBANS' total score and subscores. However, since eye‐tracking parameters were abnormal for all participants tested, and no control group was included, it is difficult to make conclusive statements on the association of the Norrbottnian GD3 patients' neurocognitive and the role of gaze paralysis.

GD is strongly associated with Parkinson's disease (PD).^33^, ^34^ Cognitive impairment in PD is associated with various genetic factors,^35^ and *GBA* mutations, particularly L444P, correlate with greater impairment in working memory, executive function, and visuospatial ability.^36^ *GBA* mutations leading to GCase activity reduction and lysosomal dysfunction have been shown, both in vivo and in vitro, to be related to increased levels of soluble and aggregated α‐synuclein. Interestingly, the accumulation of α‐synuclein results in a reduction of GCase activity, the mechanism, though, is not entirely understood.^37^ Although PD and GD are two distinct disease entities, studies on the role of *GBA* in well‐described disease cohorts like the Norrbottnian GD3 patients could provide important insight into its function in disease processes.

Our study has several limitations, the most important of which is the small number of patients that prevents the generalizability of our findings. Also, the RBANS test does not provide a thorough neuropsychological evaluation rather than an overview of the patients' cognitive profile. However, it was considered appropriate given the practical difficulties of more time‐consuming neurocognitive evaluation in patients who suffer physical limitations. Our results are in line with previous studies on GD3 patients but in contrast to most of the previous published data, it includes adult patients of a GD3 subgroup (i.e., the Norrbottnian subtype of GD3).

During the past few years, there have been attempts to build registries of neuronopathic GD patients to better describe the natural course of the disease as well as the effect of different types of treatment. Early cognitive and neurological screening and longitudinal assessment with standardized tools in large‐size, multicentre cohort studies will provide valuable information on the evolution and treatment of CNS manifestations in neuronopathic GD.

In conclusion, RBANS appears to be a useful tool to assess the cognitive profile of Norrbottninan GD3 patients. However, more studies where the results of RBANS would be compared to those of a detailed neurocognitive evaluation are needed in order to examine whether the two assessments would be in accordance. RBANS has indicated that Norrbottnian GD3 patients show a unique neurocognitive profile with low overall performance, mostly derived from low scores in attention and memory domains whereas language and visuospatial/constructional ability are intact.

## CONFLICT OF INTEREST

Panagiota Tsitsi has received reimbursement from Parkinsonfonden, for attending the European Academy of Neurology Congress in Lisbon, 2018 in order to present preliminary results of the study. Panagiota Tsitsi and Per Svenningsson are investigators in the MOVES‐PD study sponsored by Sanofi‐Genzyme. Per Svenningsson and Maciej Machaczka have received honoraria for lecturing on Gaucher disease from Sanofi‐Genzyme and Shire/Takeda. The other authors declare that they have no significant competing financial, professional, or personal interests that might have influenced the performance or presentation of the work described in this manuscript.

## AUTHOR CONTRIBUTIONS

*Research Project: Conception, Design, Execution; Statistical Analysis: Design, Execution, Analysis and Interpretation; Manuscript Preparation: Writing of the First Draft, Review and Critique, Drafting Tables and/or Figures*: Panagiota Tsitsi. *Research Project: Conception, Design; Statistical Analysis: Execution, Analysis and Interpretation; Manuscript Preparation: Review and Critique, Drafting Tables and/or Figures*.: Ioanna Markaki. *Research Project: Conception, Design; Statistical Analysis: Analysis and Interpretation; Manuscript Preparation: Review and Critique, Drafting Tables and/or Figures*: Josefine Wardthaler. *Research Project: Conception, Design; Statistical Analysis: Analysis and Interpretation; Manuscript Preparation: Review and Critique, Drafting Tables and/or Figures*: Maciej Machaczka. *Research Project: Conception, Design, Execution; Statistical Analysis: Analysis and Interpretation; Manuscript Preparation: Review and Critique, Drafting Tables and/or Figures*: Per Svenningsson. Corresponding Author and Guarantor: Panagiota Tsitsi is guarantor for the article and accepts full responsibility for the work and the conduct of the study, had access to the data, and controlled the decision to publish.

## ETHICS STATEMENT

All procedures followed were in accordance with the ethical standards of the local ethics committee (Regionala Etikprövningsnämnden Stockholm), which approved the study (DNR 2016/19‐31/1 and 2017/1957‐32/1), and in accordance with the Helsinki Declaration of 1975, as revised in 2000. All participants provided oral and written informed consent. Proof that informed consent was obtained is available upon request. This article does not contain any studies with animal subjects performed by the any of the authors.

## Data Availability

The datasets generated and analyzed during this study are available from the corresponding author on reasonable request.

## References

[1] Roshan Lal T , Sidransky E . The spectrum of neurological manifestations associated with gaucher disease. Diseases. 2017;5:10. 10.3390/diseases5010010 PMC5456331 28933363

[2] Stirnemann J , Belmatoug N , Camou F , et al. A review of Gaucher disease pathophysiology, clinical presentation and treatments. Int J Mol Sci. 2017;18:441. 10.3390/ijms18020441 PMC5343975 28218669

[3] Schiffmann R , Sevigny J , Rolfs A , et al. The definition of neuronopathic Gaucher disease. J Inherit Metab Dis. 2020;43:1056‐1059. 32242941 10.1002/jimd.12235 PMC7540563

[4] Dreborg S , Erikson A , Hagberg B . Gaucher disease—Norrbottnian type I. General clinical description. Eur J Pediatr. 1980;133:107‐118. 7363908 10.1007/BF00441578

[5] Blume J , Beniaminov S , Kämpe Björkvall C , Machaczka M , Svenningsson P . Saccadic impairments in patients with the norrbottnian form of Gaucher's disease type 3. Front Neurol. 2017;8:295. 28690585 10.3389/fneur.2017.00295 PMC5479920

[6] Machaczka M , Paucar M , Bjorkvall CK , et al. Novel hyperkinetic dystonia‐like manifestation and neurological disease course of Swedish Gaucher patients. Blood Cells Mol Dis. 2018;68:86‐92. 27789132 10.1016/j.bcmd.2016.10.011

[7] Machaczka M , Kämpe Björkvall C , Wieremiejczyk J , et al. Impact of imiglucerase supply shortage on clinical and laboratory parameters in norrbottnian patients with Gaucher disease type 3. Arch Immunol Ther Exp (Warsz). 2015;63:65‐71. 25205209 10.1007/s00005-014-0308-8 PMC4289531

[8] Dahl N , Lagerström M , Erikson A , Pettersson U . Gaucher disease type III (norrbottnian type) is caused by a single mutation in exon 10 of the glucocerebrosidase gene. Am J Hum Genet. 1990;47:275‐278. 2378352 PMC1683716

[9] Van Rossum A , Holsopple M . Enzyme replacement or substrate reduction? A review of Gaucher disease treatment options. Hosp Pharm. 2016;51:553‐563. 27559188 10.1310/hpj5107-553 PMC4981103

[10] Erikson A , Forsberg H , Nilsson M , Aström M , Månsson JE . Ten years' experience of enzyme infusion therapy of norrbottnian (type 3) Gaucher disease. Acta Paediatr. 2006;95:312‐317. 16497642 10.1080/08035250500423804

[11] Wong K , Sidransky E , Verma A , et al. Neuropathology provides clues to the pathophysiology of Gaucher disease. Mol Genet Metab. 2004;82:192‐207. 15234332 10.1016/j.ymgme.2004.04.011

[12] Orvisky E , Park JK , LaMarca ME , et al. Glucosylsphingosine accumulation in tissues from patients with Gaucher disease: correlation with phenotype and genotype. Mol Genet Metab. 2002;76:262‐270. 12208131 10.1016/s1096-7192(02)00117-8

[13] Bouscary A , Quessada C , René F , et al. Sphingolipids metabolism alteration in the central nervous system: amyotrophic lateral sclerosis (als) and other neurodegenerative diseases. Semin Cell Dev Biol. 2021;112:82‐91. 33160824 10.1016/j.semcdb.2020.10.008

[14] Sipione S , Monyror J , Galleguillos D , Steinberg N , Kadam V . Gangliosides in the brain: physiology, pathophysiology and therapeutic applications. Front Neurosci. 2020;14:572965. 33117120 10.3389/fnins.2020.572965 PMC7574889

[15] Ito S , Barrett AJ . Gauchers disease—a reappraisal of hematopoietic stem cell transplantation. Pediatr Hematol Oncol. 2013;30:61‐70. 23363328 10.3109/08880018.2012.762076

[16] Erikson A , Karlberg J , Skogman AL , Dreborg S . Gaucher disease (type III): intellectual profile. Pediatr Neurol. 1987;3:87‐91. 3508057 10.1016/0887-8994(87)90033-6

[17] Steward AM , Wiggs E , Lindstrom T , et al. Variation in cognitive function over time in Gaucher disease type 3. Neurology. 2019;93:e2272‐e2283. 31719137 10.1212/WNL.0000000000008618 PMC6937490

[18] Davies EH , Mengel E , Tylki‐Szymanska A , Kleinotiene G , Reinke J , Vellodi A . Four‐year follow‐up of chronic neuronopathic Gaucher disease in europeans using a modified severity scoring tool. J Inherit Metab Dis. 2011;34:1053‐1059. 21626202 10.1007/s10545-011-9347-z

[19] Randolph C , Tierney MC , Mohr E , Chase TN . The repeatable battery for the assessment of neuropsychological status (rbans): preliminary clinical validity. J Clin Exp Neuropsychol. 1998;20:310‐319. 9845158 10.1076/jcen.20.3.310.823

[20] Holden HM , Milano NJ , Horner MD . Five‐factor structure of the rbans is supported in an Alzheimer's disease sample: implications for validation of neuropsychological assessment instruments. Appl Neuropsychol Adult. 2020;27:232‐242. 30380924 10.1080/23279095.2018.1529671

[21] Alosco ML , Gunstad J , Jerskey BA , et al. The adverse effects of reduced cerebral perfusion on cognition and brain structure in older adults with cardiovascular disease. Brain Behav. 2013;3:626‐636. 24363966 10.1002/brb3.171 PMC3868168

[22] Kringle EA , Terhorst L , Butters MA , Skidmore ER . Clinical predictors of engagement in inpatient rehabilitation among stroke survivors with cognitive deficits: an exploratory study. J Int Neuropsychol Soc. 2018;24:572‐583. 29552996 10.1017/S1355617718000085 PMC6035068

[23] Goker‐Alpan O , Wiggs EA , Eblan MJ , et al. Cognitive outcome in treated patients with chronic neuronopathic Gaucher disease. J Pediatr. 2008;153:89‐94. 18571543 10.1016/j.jpeds.2007.12.023

[24] Vellodi A , Tylki‐Szymanska A , Davies EH , et al. Management of neuronopathic Gaucher disease: revised recommendations. J Inherit Metab Dis. 2009;32:660‐664. 19655269 10.1007/s10545-009-1164-2

[25] Tantawy AA , Sherif EM , Adly AA , Hassanine S , Awad AH . Evoked potentials and neurocognitive functions in pediatric Egyptian Gaucher patients on enzyme replacement therapy: a single center experience. J Inherit Metab Dis. 2013;36:1025‐1037. 23508695 10.1007/s10545-013-9597-z

[26] Ceravolo F , Grisolia M , Sestito S , Falvo F , Moricca MT , Concolino D . Combination therapy in a patient with chronic neuronopathic Gaucher disease: a case report. J Med Case Reports. 2017;11:19. 10.1186/s13256-016-1147-5 PMC5248516 28103924

[27] Cox‐Brinkman J , van Breemen MJ , van Maldegem BT , et al. Potential efficacy of enzyme replacement and substrate reduction therapy in three siblings with Gaucher disease type iii. J Inherit Metab Dis. 2008;31:745‐752. 18850301 10.1007/s10545-008-0873-2

[28] Machaczka M . Allogeneic hematopoietic stem cell transplantation for treatment of Gaucher disease. Pediatr Hematol Oncol. 2013;30:459‐461. 23647506 10.3109/08880018.2013.793757

[29] Sharafeldin N , Bosworth A , Patel SK , et al. Cognitive functioning after hematopoietic cell transplantation for hematologic malignancy: results from a prospective longitudinal study. J Clin Oncol. 2018;36:463‐475. 29252122 10.1200/JCO.2017.74.2270

[30] Elstein D , Abrahamov A , Altarescu G , Zimran A . Evolving features in type 3 Gaucher disease on long‐term enzyme replacement therapy. Blood Cells Mol Dis. 2013;50:140. 23085428 10.1016/j.bcmd.2012.09.008

[31] Botross NP , Riad AA , Viswanathan S , Nordin RB , Lock HN . Chronic neuronopathic type of Gaucher's disease with progressive myoclonic epilepsy in the absence of visceromegaly and bone involvement. Scott Med J. 2014;59:e1‐e6. 10.1177/0036933014529868 24671628

[32] Helmstaedter C , Witt JA . Epilepsy and cognition—a bidirectional relationship? Seizure. 2017;49:83‐89. 28284559 10.1016/j.seizure.2017.02.017

[33] Bultron G , Kacena K , Pearson D , et al. The risk of Parkinson's disease in type 1 Gaucher disease. J Inherit Metab Dis. 2010;33:167‐173. 20177787 10.1007/s10545-010-9055-0 PMC2887303

[34] Sidransky E , Nalls MA , Aasly JO , et al. Multicenter analysis of glucocerebrosidase mutations in Parkinson's disease. N Engl J Med. 2009;361:1651‐1661. 19846850 10.1056/NEJMoa0901281 PMC2856322

[35] Fagan ES , Pihlstrom L . Genetic risk factors for cognitive decline in Parkinson's disease: a review of the literature. Eur J Neurol. 2017;24:561‐e520. 28220571 10.1111/ene.13258

[36] Mata IF , Leverenz JB , Weintraub D , et al. Gba variants are associated with a distinct pattern of cognitive deficits in Parkinson's disease. Mov Disord. 2016;31:95‐102. 26296077 10.1002/mds.26359 PMC4724255

[37] Muñoz SS , Petersen D , Marlet FR , Kücükköse E , Galvagnion C . The interplay between glucocerebrosidase, α‐synuclein and lipids in human models of Parkinson's disease. Biophys Chem. 2021;273:106534. 33832803 10.1016/j.bpc.2020.106534

